# No regrets: Young adult patients in psychiatry report positive reactions to biobank participation

**DOI:** 10.1186/s12888-017-1199-0

**Published:** 2017-01-17

**Authors:** Janet L. Cunningham, Manuel Zanzi, Mimmie Willebrand, Lisa Ekselius, Mia Ramklint

**Affiliations:** Department of Neuroscience, Psychiatry, Uppsala University Hospital, Entrance 10, Floor 3B, 751 85, Uppsala, Sweden

**Keywords:** Biobanking, Disability, Ethics, General psychiatry, Voluntariness

## Abstract

**Background:**

Research in vulnerable individuals must insure voluntariness and minimize negative reactions caused by participation. This study aimed to describe consent and completion rate in young psychiatric patients in relation to study components, degree of disability and to compare response to research participation in patients and controls.

**Methods:**

Between 2012 and 2015, 463 patients with psychiatric disorders between the ages of 18–25 from the Dept. of General Psychiatry at Uppsala University Hospital and 105 controls were recruited to donate data and samples to a biobank. Consent and completion in relation to questionnaires, biological sampling of blood, saliva or feces, were monitored. Both groups were also asked about their perceived disability and how research participation affected them.

**Results:**

Most patients who participated consented to and completed questionnaires and blood sampling. The majority also consented to saliva sampling, while less than half consented to collect feces. Of those who gave consent to saliva and feces only half completed the sampling. Both patients and controls reported high voluntariness and were positive to research participation. Within the patient group, those with greater perceived disability reported greater distress while participating in research, but there was no difference in consent or completion rates or level of regret.

**Conclusions:**

With the described information procedures, psychiatric patients, regardless of perceived disability, reported high voluntariness and did not regret participation in biobanking. Compared to questionnaires and blood sampling, given consent was reduced for feces and completion was lower for both saliva and feces sampling.

## Background

Current clinical practice in psychiatry is conducted through subjective evaluation of phenotypes. Diagnostic instruments, such as structured interviews and questionnaires, greatly improve the sorting of patients into valid diagnostic groups for generalizations about appropriate treatment. Biological markers are, however, absent and an important dimension of diagnostics and basis for understanding disease etiology is missing. Recent research suggest, for example, that interconnected links between the immune system, the microbiome and the brain are essential for normal brain functions, such as initiating and regulating stress responses, emotions and behavior [1, 2]. The next step is to define clinical markers based on integrated data that reflect biological pathways and look for meaningful clinical differences in large representative patient cohorts. Systematic collection of biological samples from patients within psychiatry are rare in contrast to other medical specializations where biobanks provide a foundation for excellent research with considerable potential to inform improved patient care. To provide a valid basis for psychiatric research, sample collection must reflect the population and include patients with significant deficits in executive function, cognitive abilities and emotional regulation. Complicated ethical aspects are a central factor contributing to the slow development within this area [3].

In 2012, our research group established an infrastructure, Uppsala Psychiatric Patient Samples (UPP), to enable systematic collection of data and biological material from young adult patients newly remitted for ambulatory psychiatric care. The data includes physical examination and questions on sensitive topics such as experience of physical and emotional trauma, self-harm, alcohol, and illicit drug abuse. The biological material includes blood tests, saliva collection and feces samples. In the planning stages of the project, there was concern about the feasibility and the ethical aspects of the project. One concern was that there were too many questions and that it would be difficult for patients to complete the full protocol. Participation in the biological sampling could be reduced by several factors, such as anxiety, blood phobia, integrity concerns or low executive functioning. Concern was raised that the questions could cause emotional distress, that the patients would feel obliged to partake or that patients would regret participation.

It is important to understand and minimize the distress that research may cause, more so in individuals considered vulnerable. The American National Bioethics Advisory Commission views the population with psychiatric disorders as a ‘vulnerable’ one, but also considers that not all people with psychiatric disorders are ‘vulnerable’ at all times and in all areas. Following this argument Yanos et al. discusses the vulnerability among persons with psychiatric disorders as a state, rather than a trait, and presents evidence that vulnerability must be evaluated in two dimensions: capacity and voluntariness, which must be considered while designing a study [4]. Capacity is the degree to which participants are able to comprehend informed consent. Voluntariness is related to the power imbalance between investigator and subject and is the act of free will with no coercion in the recruitment of participants. Voluntariness is also dependent on the ability to withdraw from the study at any time [5]. Both capacity and voluntariness are extra important in the case of studies based on biological samples from a biobank. In this study, a broad consent approach was selected as the most suitable option. This consent involves a broad a decision to allow others to decide based on general information that the future use of the biobank samples would be used in research that meet proper ethical and confidential standards [6, 7].

In a community-based study with a standardized psychiatric interview [8], a small minority, 2.7%, reported the interviews as quite distressing. In patients with psychiatric disorders 9% reported anxiety after interview study participation [9]. A review article summarizes that a small minority experience distress during psychiatric research, while distress is more common in studies examining trauma [10]. A trauma-related study finds that despite distress caused by participation, the experience as a whole was viewed as positive and interesting and this held true even for patients who were highly symptomatic for posttraumatic stress disorder (PTSD) [11]. Further, ‘vulnerable’ individuals, in various domains (economic, social, psychological, and physical health) required to give a small blood sample through finger pricks, had stronger emotional reactions to research participation than less vulnerable but were not less willing to continue participating [12].

To evaluate ethical and feasibility issues related to psychiatric biobanking, this study aimed to describe consent and completion rate in young psychiatric patients in relation to study component; questionnaires or biological samples and in relation to degree of disability and to compare response to research participation in patients with controls. We hypothesized that patients are more likely than healthy controls to report negative emotions in response to research, however, they are more likely to report research participation as meaningful, and equal or less likely to regret participation. We also hypothesized that degree of disability in psychiatric patients would correlate negatively with consent and completion rates, and positively with degree of distress caused by research participation.

## Methods

### Participants

#### Study population characteristics

Between August 2012 and September 2015, all new patients, 18–25 years of age, with primarily affective and anxiety disorders, receiving ambulatory care at the “Young Adults” section of Dept. of General Psychiatry at Uppsala University Hospital were asked to participate in Uppsala Psychiatric Patient Samples (UPP). The phase and degree of illness was highly variable. Some patients were remitted from other medical units already in stabilized in treatment while others were untreated. The large majority of patients reported current symptoms of depression and/or anxiety. In total, 1119 patients were approached and of them 463 (41.4%) accepted to participate. For those who gave consent, diagnoses were taken from the medical records (457 patients). Table 1 shows the participant characteristics of the patient group.

**Table 1:** Characteristics of participants who chose to participate in UPP

		*n* (%)
Patients		
	Total (%)	463 (100)
Age	Mean (range)	21 (18–25)
Gender		
	Female	362 (78.2)
	Male	101 (21.8)
Diagnosis (current)		
	Any major depressive disorder	134 (67.8)
	Any bipolar disorder	58 (12.5)
	Any anxiety disorder	278 (60.0)
	Missing information	6 (1.3)
Level of education		
	University	211 (45.6)
	Upper secondary school	191 (41.3)
	9 year elementary school	31 (6.7)
	Missing information	10 (2.2)
Controls		
	Total (%)	105 (100)
Age		22 (18–30)
Gender		
	Female	79 (75.2)
	Male	26 (24.8)
Diagnosis (current)		
	Any major depressive disorder	0
	Any bipolar disorder	0
	Any anxiety disorder	5 (4.7)
	Missing information	5 (4.7)
Level of Education		
	University	101 (96.2)
	Upper secondary school	0
	9 year elementary school	1 (0.01)
	Missing information	3 (0.03)

A control group was recruited from university employees and students. Controls were paid a modest sum (250 SEK) to be subject to the same procedures and questionnaires as the patient group (see below). The control group had an age range from 18 to 60, but for the purpose of this paper we have included those between ages 18–30 to better match the patient group (*n* = 105). Table 1 shows the characteristics of the control group.

### Participant recruitment and informed consent

A research assistant personally informed patients with oral and written information about UPP, answered questions and acquired signed consent. All patients were informed in writing that i) their participation was voluntary and would not influence their treatment; ii) samples would be used for research on biological mechanisms related to psychiatric disease and treatment and several examples of possible research questions were given; iii) new research questions using the samples must be approved by the ethical comities and that new consent from the patients may or may not be required; iv) that coded samples may be sent to international laboratories and data published in closed and open databases; v) The identity of the participants is protected and not available to persons outside the research group and that all researches working with the data are bound by the same confidentiality laws as hospital employees vi) patients may once a year request their data and results free of charge; vii) patients may leave the study at any time point without providing an explanation and that the data and samples would remain in the study as anonymous. All patients were asked to sign a broad consent to research that identify diagnostic biological markers (including genetic, hormonal, intestinal microflora and inflammatory markers) for disease to identify differences between diagnostic groups, and to follow biological changes during treatment. Patients were also asked specifically to consent by checking a box to each of the following: collection of diagnostic information from medical records, undergo thorough clinical assessment (questionnaires and interview), physical examination, blood test for analysis of hormones and changes in the immune system and genetic analysis, repeated saliva samples during a day, an adrenal function (dexametasone) test, and in case of severe symptoms consent to lumbar puncture, finally to consent to contact in the future for monitoring and sampling.

The consent form was modified in 2013 and two questions were added, asking for consent to collect information in national health and population registers and the specific consent with a check box to provide feces, since this was included among the biological samples but not separately specified in the first form. Feces sampling was reported by patients as more troublesome than blood and saliva sampling and the modified consent form allowed a post-hoc analysis of participants’ capacity to decline participation. Furthermore, the consent to collect lumbar fluid was modified so that patients would better understand when this sampling would be applicable, namely when symptom grade indicated clinical usage of lumbar fluid for diagnostics. In a post-hoc analysis, consent and completion rates were compared before and after modifications were introduced in the consent form to assess the impact of these changes on the decision to participate.

The patient population had the option to opt out of any of these procedures, while, for the sake of complete data, the control group was asked to participate in all of them, with the exception of the collection of cerebrospinal fluid sample, however this did not affect their ability to withdraw from the study at any time. Controls were recruited by oral and written information among students and employees at the university. The same research assistant as for the patients answered questions, acquired signed consent and conducted the investigation. The written consent form for controls was shorter and less detailed and participants were not given the option to opt out of sections of the study.

### Instruments

The questionnaires and the topics addressed are presented in Table 2.

**Table 2:** Study components for which specific consent was requested, instruments and details about the method, number of items or time to complete

Study Component	Instruments	Details
*Clinical data*		
Diagnostic interview and clinical data	Structured Clinical Interview for DSM-IV Axis I Disorders- Clinical Version [21] or Mini-International Neuropsychiatric Interview [22, 23] from medical records	30–60 min
Clinical examination	Height, weight, waist-hip measurement, blood pressure, pulse.	15 min
*Biological samples*		
Blood	EDTA-blood and serum 25 ml	Conducted at the clinic
Saliva	6 samples over 1 day	Conducted at home
Feces sampling	1 ml sample	Conducted at home
Lumbar fluid^b^	10 ml	Conducted at clinic
Adrenal function test^b^	Dexametasone test	Conducted at home
*Questionnaires*		
Alcohol and drug use	Alcohol Use Disorders Identification Test and Drug Use Disorders Identification Test [24]	21 items
Depressive symptoms	Montgomery Åsberg Depression Rating Scale [25–27]	9 items
Personality	Swedish universities Scales of Personality [28]	91 items
Disability	The Sheehan Disability Scale [29, 30]	5 items
Self harm	Deliberate Self Harm Inventory: 9-Item Version [31]	9 items
Sleep quality	Pittsburgh Sleep Quality Index [32]	19 items
Traumatic life events	Early Trauma Inventory [33]	62 items
Sociodemografics, medical history, heredity, menstruation patterns in women, pain	Questions identical to those used in LifeGene (http://www.lifegene.se) and EpiHealth (http://www.epihealth.se)	30 items
Gastrointestinal symptoms	The Gastrointestinal Symptom Rating Scale [34]	15 items
Participant reactions	Response to Research Participation Questionnaire [13, 35]	12 items
*Follow-up*		
National registers^b^	Registers concerning cause of death, disease, pharmaceutical usage	
Re-contact^b^	Permission to contact follow-up.	

^a^Obtained only for the patient group. ^b^Not initiated

### Reactions to Research Participation Questionnaire (RRPQ)

RRPQ is an adaptation of the parent version, RRPQ-P [13]. The items are not specific to any particular group of research participants. The RRPQ-P was translated with permission by the originators by three independent researchers (among them authors MR and MW) and a bilingual native English-speaking translator conducted a back translation. There was only minor divergence and the final version was decided by consensus. In the Swedish version, 12 items were included. Items are rated from 1 = “I do not agree at all”, to 5 = “I agree completely”. The scores for the three negative appraisal items (Q1, 4 and 6) were reversed before analysis. RRPQ was administered after blood sampling as the last questionnaire. Feces and saliva tests had in some cases not been completed.

### Sheehan Disability Scale (SDS)

To measure perceived disability SDS [14] was used. SDS is a brief self-report tool, with three items, where the study subject rates the extent to which school/work, social life and home/family life is impaired by the symptoms. It’s a valid, reliable measure of disability when used in participants with psychiatric disorders [15–17].

### Statistics

Statistics were performed using SPSS software version 21. Data were estimated to be normally distributed if it fulfilled two conditions; Skewness ratio (skewness divided by the standard error of skewness between ± 2); visually estimated normal distribution (histogram). SDS in patients fulfilled assumptions of normality, but in the control group, the SDS total and subscales and RRPQ-P items in both groups were skewed. Group differences in the individual RRPQ-P items were tested using Mann-Whitney U test (mean rank). The correlations between completion of saliva and feces collection and the SDS were tested. The controls were not included in these results, as they could not choose to opt-out of specific study components.

Correlations were tested using Spearman’s bivariate rank correlation. Statistical significance was defined at *p* < 0.05.

## Results

### Participation rates

Table 3 shows how the patients chose to consent to participation in the different areas. The majority of patients consented to access to medical records, health examination and questionnaires as well as blood sampling. Among those 95.9% who consented to blood tests, only four patients, 0.9%, declined genetic analyses of these samples. Consent for saliva collection was also high. When the specific question concerning feces was added in the consent form, consent was lower than for saliva, 58% vs. 90.5%.

**Table 3:** Consent and completion rates for patients in Uppsala Psychiatric Patient Samples (UPP)

	Consent n (%)	Completed n (%^a^)
ASKED TO PARTICIPATE IN UPP	1119 (100)	
Declined to participate	656 (58.6)	
PARTICIPANTS IN UPP	463 (41.4)	
Included in UPP	463 (100)	n/a
Access to medical records	457 (98.5)	457 (100)
Questionnaires and health examination	460 (99.1)	460 (100)
Blood test, hormone	445 (95.9)	435 (97.6)
Blood test, genetics	441 (95.0)	435 (98.6)
Saliva	420 (90.5)	202 (47.9)
Feces (*n* = 299)^b^	n/a	111 (37)
Feces (*n* = 164)^b^	98 (59.8)^b^	47(48)
Adrenal Function	387 (83.4)	ni
Lumbar puncture (*n* = 299)	134 (44.8)^b^	ni
Lumbar puncture (n = 164)^b^	118 (72)^b^	ni
Collection of information in the national health and population registers (*n* = 164)	156 (94)^b^	ni
To be contacted in the future	442 (95.2)	ni

*ni* Not been implemented. ^a^Percentage of study participants who consented and completed test presented. ^b^Number of eligible participants is lower due to a modified consent form after the first 298 study participants

#### Post-hoc analysis of participation rates in relation to modifications in consent forms

Completion rates were high for questionnaires and blood sampling, but not for saliva and feces. The modification of the consent form modification from a general consent to a specific check-box for consent for feces donation resulted in lower completion. Initially, when feces were collected in conjunction with saliva without explicit consent, completion for feces was 37% among participants. When the consent form was modified with a checkbox for feces collection, completion was 48% among those who consented, which is similar to saliva. Completion for feces in relation to the whole population (consenting and non-consenting) fell to 29% after this modification.

The modification of the consent form to specify that lumbar puncture applied only when symptom grade indicated clinical usage of lumbar fluid for diagnostics, led to a higher consent rate from 45.0 to 71.1%. Analysis of completion rates was not conducted for lumbar puncture, adrenal function test, information collection in national registers and future contact as these have not yet been implemented.

### Reactions to research participation in patients and controls

Complete data for RRPQ-P was available for 405 patients and 93 controls. Item scores ranged from 1 to 5. Median scores in all cases were equal or greater than 4. See Table 4 for answers on the RRPQ, both patients and controls generally rated their experience as very positive. Difference between patients and controls in mean rank scores for individual questions were tested and the only significant difference was in “I was told the truth about study” and “Knew I could skip questions” where patients score higher than controls (z =−4.52, *p* < 0,001 and z = −5.24, *p* < 0.001 respectively).

**Table 4:** Mean response to each question on the Reactions to Research Participation Questionnaire in patients and controls

Brief item description	Patients		Controls		Z
	Mean	SD	Mean	SD	
Q1 Being in the study was boring^a^	4.26	0.89	4.24	0.76	−0.71
Q2 Glad I was in this study	4.23	0.84	4.15	0.81	−1.13
Q3 My choice (could have said no)	4.82	0.59	4.85	0.53	−0.21
Q4 Participation made me feel sad/upset^a^	4.59	0.78	4.71	0.66	−1.510
Q5 The things I said will stay private	4.29	0.98	4.47	0.93	−1.96
Q6 Sorry I was in this study^a^	4.90	0.34	4.89	0.32	−0.629
Q7 Feel good about self	3.57	1.18	3.81	0.81	−1.28
Q8 Was told truth about study	4.71	0.65	4.38	0.86	−4.53***
Q9 Feel good about helping others	4.68	0.72	4.68	0.62	0.68
Q10 Knew I could skip questions	4.13	1.21	3.30	1.48	−5.24***
Q11 Knew I could stop at any time	4.62	0.82	4.74	0.72	−1.67
Q12 Knew I could take a break	4.32	1.06	4.11	1.26	−1.36

^a^reversed score ***p < 0.0001

Patients who donated feces before and after changes in the consent form were compared for each question on the RRPQ-P. Interestingly, despite generally positive scores for both groups, there was significant difference in question “I was glad I was in this study”, where patients who gave specific consent for feces sampling had higher mean score (4.42 ± 0.81 vs. 4.15 ± 0.85, z = −3.46; *p* < 0.001). Patients who gave consent for feces sampling but did not follow through gave higher score for the question “I chose to participate” than those who did complete feces sampling (mean score 4.93 ± 0.33 vs 4.63 ± 0.93; z = −2.95, *p* < 0.01).

### Disability in psychiatric patients in relation to consent and completion

Patients rated their disability higher than controls, total SDS score 17.0 vs. 2.9 (z = −13.24, *p* < 0.001), and for specific scales; school/work 5.98 vs. 0.99 (z = −13.16, *p* < 0.001), social life 5.86 vs. 1.05 (z = −12.89, *p* < 0.001) and home/family 5.35 vs. 0.87 (z = −13.58, *p* < 0.001), indicating greater potential vulnerability in patients. Analysis of patients’ disability in relation to consent to specific parts of the study showed, in contrast to the hypothesis, small positive correlations between higher degree of disability and given consent for adrenal function test (0.10 *p* < 0.05) and contact in the future (0.11, *p* < 0.05). There was no correlation between completed study components and disability as measured with SDS.

### Disability in psychiatric patients in relation to reactions to research participation

In the patient group, a correlation was found between responses that reflect higher distress on the RRPQ question, “Being in the study made me sad or upset”, and higher disability, measured as total SDS score (*r* = 0.20, *p* < 0.001), and with specific SDS subscales; school/work (*r* = 0.18, *p* < 0.001) social life (*r* = 0.19, *p* < 0.001), and home/family life, (*r* = 0.18, *p* < 0.001). No correlation was found between perceived disability and participation regret (*r* = 0.02, *p* = 0.71).

## Discussion

Psychiatric research must include vulnerable individuals in order to be valid and this involves ethical risks [3]. Young adult psychiatric patients who participated in psychiatric biobank sampling reported high voluntariness and did not regret participation. Patients did not differ from controls in this matter, despite a large difference in psychiatric symptoms and perceived disability between the groups. When individual questions were compared, the only significant difference between the groups was found for the question “I was told the truth about the study”, where although both groups had high scores, the patient group scored higher, and therefore considered themselves to be better informed, than their control counterparts. This difference may be due to differences in recruitment procedures. Before study start and continually throughout the study, ethical concerns and procedural barriers in obtaining signed consent were discussed within the research group and clinic. We believe that the ethical awareness of the research assistant contributed greatly to engaging patients at this acute stage. Monitoring subjective experience of research participation provided an important validation of the informed consent procedure applied in this setting and this method can easily be applied in other research contexts.

Patients giving consent largely participated in both questionnaires and blood tests. There was no difference in consent given for genetic compared to hormone analysis of blood, which is interesting since ethical committees often consider genetic analyses more ethically challenging. Participation in saliva and feces collection was much lower than for blood, While were are not aware of earlier studies concerning feces donation, an earlier general population survey indicates that willingness to donate saliva is higher than for blood [18]. Participation in saliva and feces collection required patients to go home with tubes and return with samples to the clinic within a limited time frame. Consent given for saliva sampling was much higher that completion. One explanation might be that the sampling method requires higher motivation and/or executive abilities. Consent for feces collection was lower than for saliva, indicating that this may be regarded as more uncomfortable. The completion rate for those who gave consent was similar to that of saliva, approximately half. Interestingly, a higher percentage of patients completed feces sampling when not specifically asked for consent. This may reflect a difference in the perceived right to choose in the two situations. It is well described that voluntariness is influenced by implicit power in the relationship between participants and investigators in research [4]. Patients not given the opportunity to decline feces sampling might have had difficulties rejecting it when they already were in the study. Accordingly, patients, who did not complete the collection of feces, although they had consented, had a stronger feeling of voluntariness than those who consented and completed. Furthermore, patients who were asked for consent were actually happier to participate than those who were not asked, indicating that more information may be negative for participation but positive for the subject’s participation experience.

Within the patient group, there was a positive correlation between the levels of distress caused by research participation and perceived disability, although there was no such correlation in the item of measuring the feeling of regret. These results are similar to earlier studies where distress was linked with vulnerability but without lesser willingness to continue with participation [12]. Additionally, perceived disability contributed to equal or higher consent and completion rates. This is encouraging as the validity of the sample collection depends on the inclusion of patients with more severe disease states and our results indicate that this can be achieved while preserving the autonomy of individual patients.

The study has several strengths, it is the largest to date concerning a general psychiatric population and includes data from controls, data was collected prospectively and diagnostic methods were standardized. The results are based on an outpatient population with primarily anxiety and affective disorders. A limitation of the study is that the results are not necessarily applicable to inpatients and/or patients with psychotic disorders. A second limitation is that data was not available on patients who declined participation in UPP from the start. Further research is needed to identify reasons behind declining participation. The Swedish population reports high levels of trust in public institutions and willingness to donate blood for research [19, 20]. Broad consent is both widely implemented and accepted in the Scandinavian countries when compared to most other European countries and therefore our findings may not be generalizable to other countries [20].

## Conclusion

The importance of evaluating the impact of research on its participants should not be understated. Ethical committees and researchers have a responsibility to both ensure high quality research and protect the participants, more so if potentially vulnerable. This study describes a method for including young adult patients seeking help for psychiatric disorders in bio-banking with broad consent. In this setting, patients report high levels of voluntariness and understanding of the information given and overwhelmingly positive participation experience. Although disability is connected to distress during research participation, it does not reduce the rate of consent or completion and patients do not regret participation. When comparing patients with a control group no significant negative difference in emotional response to research participation was found. Interest in biological mechanisms related to psychiatric disorders is growing and these results indicate that careful information and consent procedures allow for representative sample collection, voluntariness and minimal negative reactions in this vulnerable patient group.

## Abbreviations

RRPQ: Reactions to research participation questionnaire
SDS: Sheehan disability scale
UPP: Uppsala psychiatric patient samples
