# Erratum to: No regrets: Young adult patients in psychiatry report positive reactions to biobank participation

**DOI:** 10.1186/s12888-017-1228-z

**Published:** 2017-02-20

**Authors:** Janet L. Cunningham, Manuel Zanzi, Mimmie Willebrand, Lisa Ekselius, Mia Ramklint

**Affiliations:** Department of Neuroscience, Psychiatry, Uppsala University Hospital, Entrance 10, Floor 3B, 751 85 Uppsala, Sweden

## Erratum

During the production of this article [1] the authors noticed that there was a mistake in Table 1. The correct version of the table appears on the following page. The value after ’Any major depressive disorder’ has been changed from 134 to 314.

**Table 1:** Characteristics of participants who chose to participate in UPP

		*n* (%)
Patients		
	Total (%)	463 (100)
Age	Mean (range)	21 (18–25)
Gender		
	Female	362 (78.2)
	Male	101 (21.8)
Diagnosis (current)		
	Any major depressive disorder	314 (67.8)
	Any bipolar disorder	58 (12.5)
	Any anxiety disorder	278 (60.0)
	Missing information	6 (1.3)
Level of education		
	University	211 (45.6)
	Upper secondary school	191 (41.3)
	9 year elementary school	31 (6.7)
	Missing information	10 (2.2)
Controls		
	Total (%)	105 (100)
Age		22 (18–30)
Gender		
	Female	79 (75.2)
	Male	26 (24.8)
Diagnosis (current)		
	Any major depressive disorder	0
	Any bipolar disorder	0
	Any anxiety disorder	5 (4.7)
	Missing information	5 (4.7)
Level of Education		
	University	101 (96.2)
	Upper secondary school	0
	9 year elementary school	1 (0.01)
	Missing information	3 (0.03)
